# Degradation of Ochratoxin A by a UV-Mutated *Aspergillus niger* Strain

**DOI:** 10.3390/toxins14050343

**Published:** 2022-05-16

**Authors:** Dong Zou, Jian Ji, Yongli Ye, Yang Yang, Jian Yu, Meng Wang, Yi Zheng, Xiulan Sun

**Affiliations:** 1 State Key Laboratory of Food Science and Technology, School of Food Science and Technology, Synergetic Innovation Center of Food Safety and Quality Control, Jiangnan University, Wuxi 214122, China; zoudong@stu.jiangnan.edu.cn (D.Z.); jijian@jiangnan.edu.cn (J.J.); yyly0222@163.com (Y.Y.); yyang7858@163.com (Y.Y.); jiangsuyzyj@163.com (J.Y.); 2 College of Food Science and Pharmacy, Xinjiang Agricultural University, Urumqi 830052, China; 3 Institute of Quality Standards and Testing Technology, Beijing Academy of Agriculture and Forestry Sciences, Beijing 100097, China; wangm@brcast.org.cn; 4 Key Laboratory for High-Tech Research and Development of Veterinary Biopharmaceuticals, Jiangsu Agri-Animal Husbandry Vocational College, Taizhou 214122, China; zysm76@163.com

**Keywords:** Ochratoxin A, detoxification, *Aspergillus niger*, degradation products, cytotoxicity

## Abstract

Ochratoxin A (OTA) is a mycotoxin that can contaminate a wide range of crops such as grains and grapes. In this study, a novel fungal mutant strain (FS-UV-21) with a high OTA degradation rate (74.5%) was obtained from *Aspergillus niger* irradiated with ultraviolet light (15 W for 20 min). The effect of pH, temperature, and inoculation concentration on the degradation of OTA by FS-UV-21 was investigated, and the results revealed that the detoxification effect was optimal (89.4%) at a pH of 8 and a temperature of 30 °C. Ultra-performance liquid chromatography-tandem mass spectrometry was used to characterize the degraded products of OTA, and the main degraded product was ochratoxin α. Triple quadrupole-linear ion trap-mass spectrometry combined with LightSight software was used to analyze the biotransformation pathway of OTA in FS-UV-21, to trace the degraded products, and to identify the main metabolite, P1 (C_19_H_18_ClNO_6_, *m*/*z* 404). After the FS-UV-21 strain was treated with OTA, the HepG2 cellular toxicity of the degradation products was significantly reduced. For the real sample, FS-UV-21 was used to remove OTA from wheat bran contaminated by mycotoxins through fermentation, resulting in the degradation of 59.8% of OTA in wheat bran. Therefore, FS-UV-21 can be applied to the degradation of OTA in agricultural products and food.

## 1. Introduction

Ochratoxin A (OTA), one of the five most prevalent mycotoxins contaminating food and feed [1], is a secondary metabolite that is produced by *Penicillium verrucosum*, *A. ochraceus*, and *A. carbonarius* [2]. It is commonly found in a variety of foods, including cereals and cereal products, wine, tea, coffee, milk and dairy products, poultry, fish, pork and eggs, fruits and vegetables, and baby food [3,4,5,6]. OTA has been classified as a class 2B carcinogen by the International Agency for Research on Cancer, and it is nephrotoxic, hepatotoxic, and genotoxic to the health of humans and animals [7,8,9]. Therefore, OTA contamination of foods and feed should be carefully managed [10]. However, many preventive measures have been taken to avoid OTA contamination [11], and many grains are still tainted by the mycotoxin. The removal of mycotoxins from food and feed are usually achieved using physical, chemical, and biological methods [12,13,14]. Physical methods, such as heat treatment and radiation, are inefficient and can destroy the nutritional value and flavor of food [15,16]. If chemical methods are used, residual chemicals that are harmful to humans and animals may remain [15]. By contrast, biological methods, which are suitable for most solid and liquid foods, have several advantages, including low cost, high effectiveness, environmental friendliness, and minimal nutrients [16,17,18]. Therefore, biological detoxification is an important research direction.

Biological agents, such as microbial strains and biological enzymes, can detoxify OTA, and thus they have broad application prospects [16]. Earlier studies have reported that certain bacterial and yeast species [19,20,21] can degrade OTA. In addition, fungi such as *A. niger* and *A. oryzae* have detoxification properties [18,22]. However, many challenges remain for the detoxification of food and feed. For instance, detoxification efficiency is affected by environmental conditions. The degradation effects of *A. oryzae* on OTA at different temperatures were significantly different [18]. Furthermore, there is a lack of understanding for the degradation products of OTA and their biotransformation by degrading strains [10,23]. Most studies have not used real samples, which are often contaminated by mycotoxins other than OTA [24]. Therefore, it is not only important to identify efficient mycotoxin-degrading strains, but also to define the mechanism of detoxification and to use real samples [25,26]. Feeds that are susceptible to mold contamination are a good choice, such as wheat bran, which is often added to livestock feed.

As one of the most convenient and effective mutagens, ultraviolet (UV) radiation has been widely used in the production of new strains [27,28]. Mutant strains often achieve better results than native strains [29]. For example, random UV mutagenesis has increased the biodiesel production of bacteria and generated mutant yeast strains with higher fat production [30,31]. In our previous study, the non-toxigenic *A. niger* FS10 strain was demonstrated to degrade aflatoxin B1 (AFB1) and zearalenone (ZEN) [32,33,34]. Based on these findings, we screened mutant strains with significantly improved abilities to remove OTA using UV radiation, and studied their detoxification mechanisms. The aims of this study were (i) to improve the degradation rate of OTA by *A. niger* (wild type) through UV radiation, and to identify the mutant strain; (ii) to analyze the degradation products of OTA, and to infer the biotransformation process of OTA in the strain; (iii) to study the ability of the strain to degrade different concentrations of OTA and to optimize the factors; and (iv) to explore the effects of removing OTA from wheat bran by *A. niger* through fermentation, and to evaluate the possibility of applying the strain to the agricultural product.

## 2. Results and Discussion

### 2.1. Screening and Identification of the Mutant Strain FS-UV-21

**Figure 1:**
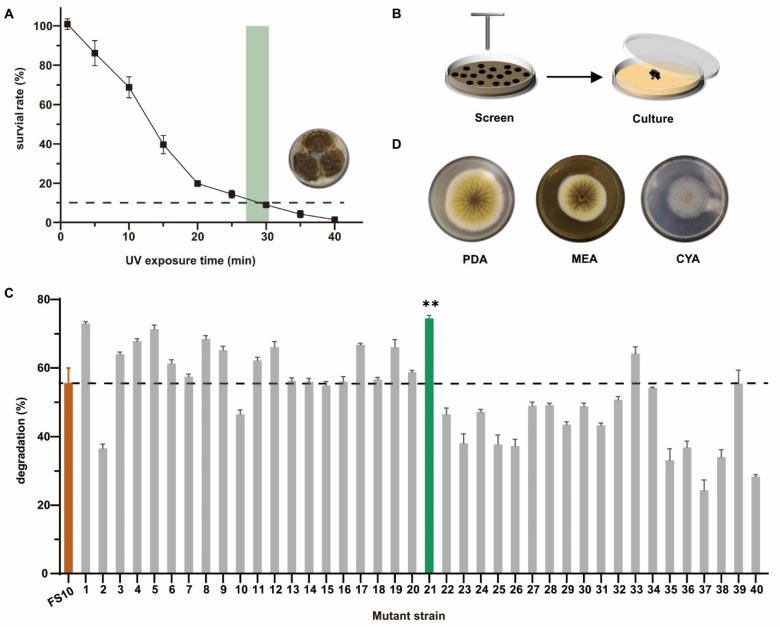
(**A**) The survival rate of the FS10 strain exposed to different UV irradiation times. (**B**) The mutant strain was selected and cultured after UV irradiation. (**C**) The degradation rate of OTA by the FS10 strain and 40 mutant strains in PDB medium (** *p* < 0.001, representing a significant difference from the control group). (**D**) The morphology of mutant strains cultured on PDA, MEA, and CYA medium for 5 d.

The *A. niger* strain FS10 is a non-toxigenic fungus that was isolated by Xu et al. [32]. Here, we improved the degradation ability of OTA by exposing FS10 to UV radiation. As shown in Figure 1A, when the UV irradiation exposure time was 28 min and the mycelial survival was approximately 10% the optimal survival rate, the mutation effect was optimal [35]. Therefore, 28 min was selected as the UV irradiation time. In total, 40 mutant *A. niger* strains were selected and cultured separately (Figure 1B). Figure 1C shows the degradation rate of OTA after treatment of the FS10 (wild type) strain and 40 mutant strains in liquid medium, and the results showed that the degradation rate of OTA of mutant strain no. 21 (FS-UV-21) was the highest (74.5%), and was significantly higher than that of the wild-type strain FS10 (55.6%). Although strains FS-UV-1 and FS-UV-5 had a similar degradation rate to FS-UV-21, they showed poor passage stability in the passage experiment, so FS-UV-21 was selected for subsequent experiments.

The morphological analysis of the FS-UV-21 strain on PDA, MEA, and CYA media revealed that this strain belonged to the species *A. niger* (Figure 1D), and these results were verified via 18S rDNA sequencing analysis (Table S1). The phylogenetic tree of FS-UV-21 is shown in Figure S1. After analyzing the sequencing results and comparing the results with the literature [36], we found a common gene, *RsaI*, indicating that the FS-UV-21 strain belonged to the species *A. niger*.

### 2.2. Degradation of OTA in Liquid Medium by FS10 and FS-UV-21

**Figure 2:**
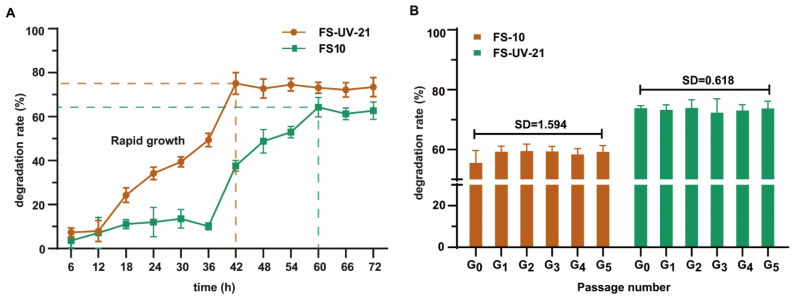
(**A**) OTA degradation rates of FS10 and FS-UV-21 strains incubated in PDB for different times. (**B**) The passage stability of *A. niger* for OTA degradation (G_0_ represents the original strain, G_1_, G_2_, G_3_, G_4_, and G_5_ represent the passage number).

Figure 2A depicts the process by which OTA was decreased in PDB medium. The degradation rate of the FS-UV-21 mutant strain was higher than that of the FS10 strain. The FS-UV-21 strain not only increased the degradation rate of OTA, but also shortened the time needed to achieve the best degradation effect. The amount of OTA in the culture medium decreased significantly over 12–48 h, which was likely due to the rapid growth of the strain, thereby increasing the cell surface area and the degradation of OTA [37,38]. Furthermore, the FS-UV-21 strain reached a plateau approximately 18 h before the FS10 strain, and a time point of 42 h was selected for subsequent experiments. The UV irradiation-exposed FS-UV-21 mutant could effectively remove OTA. To examine the degradation of OTA, the original FS-UV-21 mutant was subcultured five times, and the degradation of OTA was assessed. As shown in Figure 2B, the SD value of the degradation rate of OTA was 0.168, which illustrated that the FS-UV-21 mutant showed strong mitotic stability.

### 2.3. Detoxification of Different Concentrations of OTA by the FS-UV-21 Strain and Optimization of Conditions

**Figure 3:**
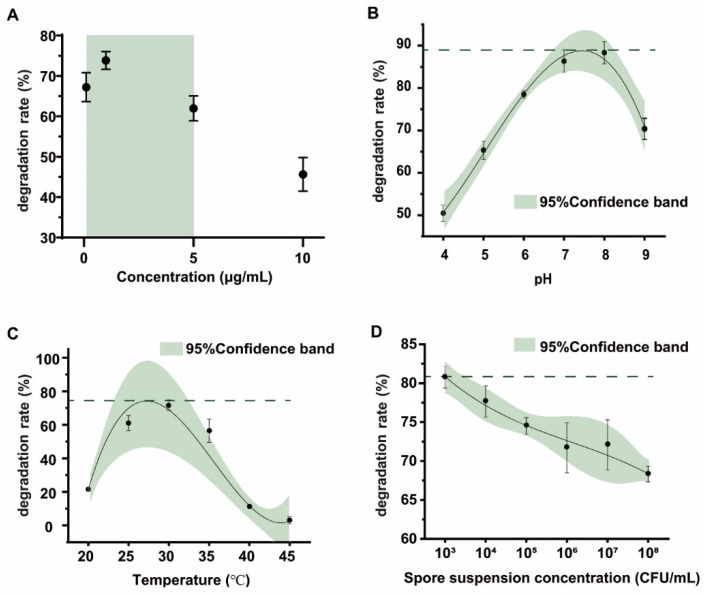
(**A**) The degradation rate of different concentrations of OTA by the FS-UV-21 strain (28 °C, 42 h). The optimization of the OTA degradation conditions by the FS-UV-21 strain: (**B**) pH (30 °C, 1 × 10^6^ CFU/mL); (**C**) Temperature (pH 6.0, 1 × 10^6^ CFU/mL); (**D**) Spore concentration (30 °C, pH 6.0).

Most biodegradation strains can effectively remove OTA at high concentrations, but the degradation effect of substrates at low concentrations is not clear [39,40]. Therefore, the degradation effect of the FS-UV-21 strain on different concentrations of OTA under the same conditions was studied. For OTA concentrations ranging from 0.1 to 5.0 μg/mL, the degradation rates were greater than 60%, which corresponded to a better biodegradation efficiency than that at concentrations that were greater than 5.0 μg/mL (Figure 3A), which may be due to the fact that high OTA concentrations have a feedback inhibitory effect on the activity of the strain. Based on a previously published study of the concentration of OTA in food, OTA contamination in most food products was less than 1 μg/mL [2]. Therefore, the FS-UV-21 strain has good application prospects.

To optimize the detoxification conditions of the FS-UV-21 strain, the effects of pH, temperature, and spore concentration on detoxification were studied. In the pH range of 4–9, the best detoxification effect was at pH 8, and the degradation rate was >88.35% (Figure 3B). The strain showed a high degree of degradation activity under alkaline conditions, indicating that pH is the key factor for the detoxification of the FS-UV-21 strain, which is consistent with the findings that OTA is relatively stable under acidic conditions, and that alkaline environments are conducive to OTA degradation [25]. However, strong alkaline environments were not conducive to the growth of the FS-UV-21 strain. As shown in Figure 3C, in the temperature range of 25–35 °C, the degradation rate of the FS-UV-21 strain was high (56.42–71.70%), and it was highest at 30 °C. This may be due to the fact that the optimal temperature had a great impact on the growth of the FS-UV-21 strain, and the degradation of OTA occurred during a period of rapid growth for the FS-UV-21 strain. Lastly, the inoculation concentration of the FS-UV-21 strain also had an effect on OTA, and the degradation of OTA (Figure 3D). When the spore suspension concentration ranged from 1 × 10^3^ to 1 × 10^8^ CFU/mL, the degradation rate of OTA decreased gradually, although insignificantly. The higher the inoculation concentration, the more metabolites were produced by *A. niger* during incubation. In practical applications, the most suitable inoculation concentration can be selected to correspond with the actual economic cost. Under a pH of 8, a temperature of 30 °C, and a spore suspension concentration of 1 × 10^3^ CFU/mL, the degradation rate of OTA by the FS-UV-21 strain was the highest at 89.36% after 42 h.

### 2.4. Identification of OTA Degradation Products

**Figure 4:**
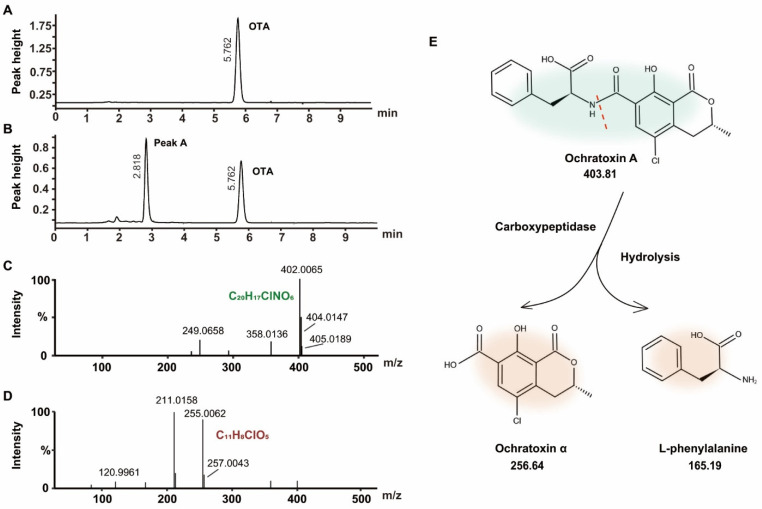
(**A**) HPLC chromatogram of the blank control group. (**B**) HPLC chromatogram of FS-UV-21 after 36 h of biological fermentation in PDB containing OTA (The retention time of OTA was 5.77 min, and that of peak A was 2.22 min). (**C**) Mass spectrum and proposed structure of OTA. (**D**) Mass spectrum and proposed structure of OTα. (**E**) Prediction of the OTA degradation process.

A comparison of the HPLC chromatograms of OTA before (Figure 4A) and OTA after exposure to the FS-UV-21 strain (Figure 4B) revealed that a new chromatographic peak (peak A) appeared at 2.818 min. The peak area of peak A increased as the peak area of OTA decreased, indicating that peak A could be the main degradation product. A previous study reported the degradation product of OTA to be ochratoxin α (OTα), especially under biodegradation or fermentation conditions [37]. The biodegradation product of OTA of *Pediococcus parvulus* isolated from Douro wines was OTα [21]. OTα was also the degradation product of OTA from exposure to *Aspergillus* [18]. Another degradation event of OTA involves lactone ring hydrolysis, but there are few studies addressing this mechanism [41]. Therefore, it can be concluded that this unknown compound was OTα.

Liquid-interface chromatography tandem mass spectrometry and quadrupole time-of-flight (UPLC-q-TOF) were utilized to identify the OTA degradation products after exposure to the FS-UV-21 strain. The OTA MS/MS spectrum is shown in Figure 4C, with a precursor ion of 402.0065[M-H]^⁻^. The MS/MS spectrum of peak A is shown in Figure 4D, with a precursor ion of 255.0062[M-H]^⁻^, and its isotope peak was 257.0043, which indicated that it contained the halogen element Cl. Based on elemental composition analysis, the elemental composition of the substance was C_11_H_9_ClO_5_, and the theoretical precursor ion [M-H]^⁻^ was 255.0066 (δMS1 = 0.0004 Da). Structural elucidation was conducted using MS Finder, and OTα had the highest score (3.4410). To study its degradation mechanism, OTA released OTα and L-phenylalanine (L-β-Phe) by hydrolyzing the OTA amide group (Figure 4E). This reaction was likely mediated by carboxypeptidase or similar enzymes [41,42]. In addition, the toxicity of OTα was low [43]. Therefore, *A. niger* could reduce the OTA content, and *A. niger* has broad application prospects in agricultural products.

### 2.5. OTA Biotransformation Pathway and Metabolic Degradation Products

De Bellis et al. [44] and Wei et al. [45] identified the biodegradation products of OTA of some strains, although they did not explore whether OTA participated in metabolic degradation. The biodegradation of OTA by the FS-UV-21 strain has been determined, but the specific metabolites of OTA and the mechanism of metabolic degradation are still unknown. According to the MS/MS spectra of OTA and OTα, the corresponding characteristic ions were *m*/*z* 358, 211, and 167, respectively. Based on the structures of OTA and its degradation products, the ion *m*/*z* 211 was selected for metabolite analysis. LightSight software can not only create up to eight methods simultaneously, but also generate metabolite information quickly and efficiently [33]. We entered the MS/MS information of OTA and selected the biotransformation pathway to automatically obtain a metabolite list containing the precursor ions (Q1) and characteristic ions (Q3) of the predicted products, as previously described [33]. Three pathways and products were analyzed (Table S2); namely, P1 (C_19_H_18_ClNO_6_, *m*/*z* 404), P2 (C_22_H_22_ClNO_8_, *m*/*z* 486), and P3 (C_19_H_16_ClNO_7_, *m*/*z* 416).

**Figure 5:**
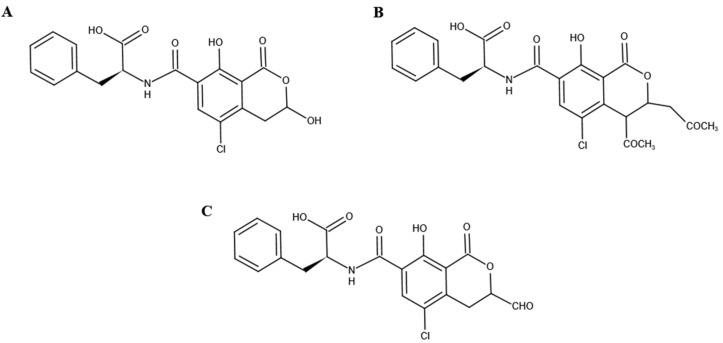
Prediction of degradation structures of OTA by the FS-UV-21 strain. (**A**) Product 1; (**B**) Product 2; (**C**) Product 3.

The inferred product structure is shown in Figure 5. An OTA analog, which has an extra methyl group compared to the structure of P1, has been reported in previous studies, but their biotransformation pathways are similar [2,46]. Therefore, P1 is the most likely transformation product of OTA in the FS-UV-21 strain. Furthermore, according to structural analysis, it is possible for P1 to transform into P3. However, the possibility of these three metabolites being converted into OTα is very small, and these metabolites may only be intermediate products. The degradation into OTα and the metabolites requires further exploration. It is also possible that these metabolites are not related to the formation of OTα, but are novel metabolites of OTA generated in *A. niger* FS-UV-21 strains.

### 2.6. Cytotoxicity Evaluation of Degradation Products

**Figure 6:**
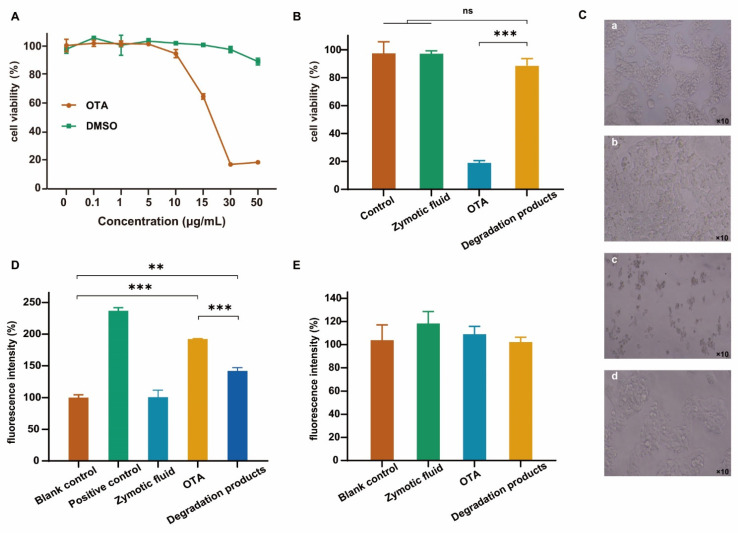
(**A**) Effects of different concentrations of OTA and an equivalent volume of DMSO on the survival rate of HepG2 cells. Effects of OTA and its metabolites on the activity and morphology of HepG2 cells; (**B**) Cell activity; (**C**) Cell morphology: (**a**) control, (**b**) zymotic fluid, (**c**) OTA, and (**d**) degradation products; (**D**) Comparison of ROS fluorescence intensity between the dose group and the blank group (fluorescence intensity was the percentage value compared with the blank control group); (**E**) Mitochondria (fluorescence intensity was the percentage value compared with the blank control group). (** *p* < 0.001, *** *p* < 0.0001, representing a significant difference from the control group; ns: not significant.).

Cell viability, intracellular ROS production, and cell morphology, which are important cytotoxicological evaluation indexes, were selected to evaluate the degradation products of OTA. OTA may induce lipid peroxidation and reduce antioxidant enzyme activity in cells [47]. The degree of cell viability induced by the OTA standard depended on the dose used (Figure 6A). According to the HepG2 cell viability e at 48 h in the presence of different OTA concentrations, the cells reached the half lethal dose (IC_50_) when the OTA concentration was 15.56 μg/mL. As such, 20 μg/mL was selected for subsequent experiments. Over a range of 0–30 μg/mL, when dimethyl sulfoxide (DMSO) was added at an equivalent volume, there was no significant difference between the cell activity and the control group, excluding the effect of DMSO on cells.

The results of the cell viability assay revealed the cytotoxicity of OTA, while the cell viability of the FS-UV-21 fermentation broth group and the degradation product group did not change significantly, compared with the control group (Figure 6B). Morphological analysis revealed no significant differences between the control group and the OTA-degraded product group (Figure 6C). The detection of intracellular ROS can directly reflect the oxidative stress of cells [48]. OTA treatment significantly increased the ROS level in HepG2 cells compared with the blank control, while the fluorescence intensity of the degradation product group increased compared with the blank control, and decreased compared with the OTA group (Figure 6D). The results showed that OTA oxidatively damaged cells, which were reduced in the FS-UV-21-treated OTA group, indicating that the degraded products had a lower cellular toxicity. The aggregation of Mito-Tracker Red CMXRos in mitochondria depends on the mitochondrial membrane potential. There was no significant difference in the fluorescence intensity among the groups (Figure 6E), indicating that OTA had little effect on the mitochondrial membrane potential of HepG2 cells.

In conclusion, the OTA fermentation product FS-UV-21 was not cytotoxic. After the FS-UV-21 strain was treated with OTA for 42 h, the cellular toxicity of the degradation products was significantly reduced. However, further animal toxicity studies are needed to gain insights into the toxicity of OTA degradation products by the FS-UV-21 strain.

### 2.7. Degradation of OTA by A. niger Biological Fermentation in Wheat Bran

Compared with the control group, the residual OTA content in wheat bran fermented with FS-UV-21 for 5 d decreased significantly (Table 1). The content of OTA in the control group was relatively stable before and after, which indicated that OTA could not be digested naturally. The FS-UV-21 strain not only degrades OTA in the culture medium, but also detoxify the wheat bran contaminated by OTA. In the future, we plan to explore whether it has the same effect on other grains. *Pediococcus parvulus* strains that were isolated from Douro wines can effectively degrade OTA in grape juice [43]. It may also be possible to inoculate some FS-UV-21 strains during the fermentation process to control the content of OTA, but attention should be paid towards whether the growth of the strain will adversely affect the quality of the wine.

## 3. Conclusions and Future Perspectives

Our study shows that the *A. niger* FS-UV-21 strain that we identified has a good degradation effect on OTA, and the highest degradation rate was 89.36% after optimizing the conditions. UPLC-q-ToF identification confirmed that the OTA degradation product degraded by *A. niger* FS-UV-21 was OTα (C_11_H_8_ClO_5_). Among the reported degradation products, OTα is considered to be non-toxic or less toxic [49,50]. Q-Trap-MS detection combined with LightSight software analysis identified three related biotransformation metabolites, in which P1 (C_19_H_18_ClNO_6_) was likely to be a new metabolite of the OTA generated through demethylation and oxidation in the FS-UV-21 strain. The results of this experiment need to be further confirmed. HepG2 cytotoxicity experiments showed that OTA not only significantly affected the morphology of HepG2 cells, but also led to decreased cell viability or even death. The toxicity of OTA to HepG2 cells was significantly reduced after FS-UV-21 treatment, which is beneficial for the safe application of FS-UV-21 in agricultural products. The application of FS-UV-21 in the fermentation of wheat bran could effectively reduce the content of OTA. However, the application of *A. niger* for degrading OTA in real samples is still in its infancy, and it cannot be directly used for OTA degradation in food. During the fermentation of wheat bran or other real samples, FS-UV-21 will improve or reduce the quality of the samples, whether these processed samples are safe as feed, and whether they will cause harm to the environment, all of which need to be verified with further experiments. Therefore, the biotransformation process of OTA needs to be further explored using nuclear magnetic resonance technology [33], and safety assessment methods are needed to determine the toxicity of the degradation products, such as better cytotoxicity assays and animal models of toxicity.

## 4. Materials and Methods

### 4.1. Microorganism and Culture Conditions

The *A. niger* FS10 strain, a non-toxigenic filamentous fungus, was identified in Chinese fermented soybean [32] and stored at the China Center for Culture Collection (CCTCC No: M2013703). Strains capable of detoxifying OTA were stored in in three solid media (PDA: potato 300 g, glucose 20 g, chloramphenicol 0.1 g, agar 20 g, distilled water 1 L, autoclaved at 121 °C for 20 min; MEA: malt extract 30 g, soybean peptone 3 g, agar 15 g, distilled water 1 L, autoclaved at 121 °C for 20 min; CYA: NaNO_3_ 3 g, KH_2_PO_4_ 1 g, KCl 0.5 g, MgSO_4_·7H_2_O 0.5 g, FeSO_4_·7H_2_O 0.01 g, yeast paste 5 g, sucrose 30 g, agar 15 g, distilled water 1 L, autoclaved at 121 °C for 20 min). To investigate OTA degradation, the isolated strains (10^6^ CFU/mL) were cultured in potato dextrose broth (PDB; potato 300 g, glucose 20 g, chloramphenicol 0.1 g, distilled water 1 L, autoclaved at 121 °C for 20 min) and incubated at 28 °C in a shaking incubator (THZ-D, Baidian, Shanghai, China) at 180 rpm for 48 h.

### 4.2. Chemicals

OTA (purity, ≥99%) were purchased from Sigma-Aldrich (St. Louis, MO, USA) and stored at −20 °C. All other analytical-grade chemicals were purchased from Sigma-Aldrich (St. Louis, MO, USA).

### 4.3. Preparation of Mutant A. niger Strains

The FS10 strain was cultured on PDA at 28 °C for 7 d. Spores were collected from PDA with sterile water containing 0.05% Tween-20 and 0.9% sodium chloride. The concentration of the spore suspension was adjusted to 10^8^ CFU/mL [35]. The *A. niger* mutant strain was obtained using UV irradiation, as described by Sun et al. [35]. In brief, FS10 was exposed to UV rays (15 W, 254 nm) for 1, 5, 10, 15, 20, 25, 30, 35, and 40 min. Thereafter, an aliquot of 20 μL of the irradiated spore suspension was cultured on PDA and incubated at 28 °C in a shaking incubator for 48 h. The unirradiated strains were used to calculate the survival rate. 

The spore suspension with a survival rate of 10% was selected and treated according to the above conditions to obtain the mutant strain. Five or six colonies were randomly selected from each plate, transferred to PDA, and cultured for 5 d. Spores were harvested. A spore suspension (10^6^ CFU/mL) was prepared with sterile water, added to PDB containing OTA (1 μg/mL) at 2% inoculum, and incubated at 28 °C in a shaking incubator for 48 h. The residual OTA content was measured to examine the ability of each mutant strain to degrade OTA. The mutant strain with the highest OTA degradation rate was used in subsequent experiments. The isolated strains were subcultured on PDA five times, and the OTA degradation rate was monitored to ensure the stability of mitosis.

### 4.4. Identification of the FS-UV-21 Strain

The 18S rDNA of the mutant strain was amplified using PCR with specific primers, and the sequence was obtained with Sequencing Analysis v5.1 software (Applied Biosystems, Wuhan, China). The sequence was compared with the deposited sequences in the GenBank database (http://blast.ncbi.nlm.nih.gov/Blast.cgi, accessed on 16 October 2020), and MEGA v7.0 software was used to calculate the sequence similarity and to construct the phylogenetic tree. FS-UV-21 was stored at the China General Microbiological Culture Collection Center.

### 4.5. Detoxification of OTA by FS10 and FS-UV-21

Spore suspensions (10^6^ CFU/mL) of the wild strain FS10 and the mutant strain FS-UV-21 were prepared as indicated, cultured on PDB containing OTA (1 µg/mL) at 2% inoculum, and incubated at 28 °C in a shaking incubator for 72 h. Samples were collected every 6 h, and the residual OTA context was measured after extraction.

### 4.6. Extraction and Analysis of OTA

The method used by Jiang et al. [51] and Bittner et al. [50] was modified to extract and detect OTA in PDB. In brief, an aliquot of 1 mL was obtained from each sample, 3 mL of chloroform acidified with 2 mol/L HCl was added, and the samples were centrifuged to obtain the lower layer solution. These steps were repeated three times. The chloroform was evaporated in a vacuum freeze dryer, re-dissolved in 60% acetonitrile, filtered with a 0.22-μm cellulose pyrogen-free filter, and used for HPLC analysis.

The extract was analyzed via HPLC (Agilent 1260, Santa Clara, CA, USA) using a C18 reversed-phase column (Thermo Hypersil Gold C18, 150 mm × 4.6 mm, 3.5 μm) and a fluorescence detector (FLD, λ exc = 333 nm and λ em = 460 nm; gain = 100). The sample was eluted at a flow rate of 1 mL/min, and the injection volume was 20 μL. The mobile phase was a mixture of acetonitrile/water/acetic acid (99:99:2, v/v/v), and the column temperature was 30 °C. 

The signal of each sample was compared with the standard concentration to determine the residual OTA concentration after incubation. The OTA standard curve was *Y* = 0.0145*X* − 0.0883, *R*^2^ = 0.9999, where *X* is the OTA concentration and *Y* is the OTA peak area detected via HPLC. Therefore, the degradation rate of OTA by the strain was calculated as follows: $Y=\frac{X1-X2}{X1}\times 100$%, where *X*1 is the concentration of OTA in the control group, *X*2 is the concentration of OTA in the treatment group, and *Y* is the degradation rate (%).

### 4.7. Effects of Different Factors on the Degradation of OTA by A. niger FS-UV-21

An OTA concentration of 1 μg/mL was selected for subsequent experiments; however, the actual concentration of OTA in contaminated products is different [2]. Therefore, different concentrations of OTA were used, namely, 10, 5, 1, and 0.1 μg/mL. The effect of incubation time on OTA degradation by FS-UV-21 has been previously examined. Therefore, the three key factors affecting the microbial degradation of OTA were selected, namely, the pH (4, 5, 6, 7, 8, and 9), temperature (20, 25, 30, 35, 40, and 45 °C), and inoculum concentration (1 × 10^3^, 1 × 10^4^, 1 × 10^5^, 1 × 10^6^, 1 × 10^7^, and 1 × 10^8^ CFU/mL). 

### 4.8. Extraction and Analysis of OTA Degradation Products

The OTA degradation products exposed to the FS-UV-21 strain and their molecular formula were analyzed using HPLC and Waters MALDI SYNAPT UPLC-q-ToF-MS. Chromatographic separation was carried out on a BEH C18 column (internal diameter, 2.1 mm × 100 mm, 1.7 μm). The mobile phase was methanol: water (45:55), the injection volume was 5 μL, the flow rate was 0.3 mL/min, and the column temperature was 30 °C. The MS conditions were as follows: negative electrospray ionization (ESI-), 4500 V ion source spray voltage, 500 °C ion source temperature, and −30 V declustering potential voltage [48]. Fragments of 255.5 and 402.0 parent ions were used for MS analysis, and the collision energy was 35 eV. To identify the compounds, the following parents to fragments were monitored: *m*/*z* 255.0→211.0 (for OTα) and *m*/*z* 402.0→358.0 (for OTA) [21]. The experimental data were analyzed and processed using Mass Lynx V4.1 software (Waters Corporation, Milford, USA).

### 4.9. QTRAP 5500 Combined with LightSight Software Analysis

Ample preparation methods for metabolite analysis were previously published by Ji et al. [52]. The detailed preparation is shown in the Supplementary Materials. The chromatographic conditions were as follows: the chromatographic column was a BEH C18 column (1.7 μm, 2.1 × 100 mm); the mobile phase A was pure water containing 0.1% formic acid, and the mobile phase B was acetonitrile; the flow rate was 350 μL/min; the injection volume was 2 μL; autosampler settings was 4 °C; and column oven temperature was 40 °C. Gradient elution is shown in Table S3. The recommended operating parameters for optimal ion source parameters were as follows: Turbo gas, 60 psi; nebulizer pressure, 60 psi; curtain gas, 35 psi; ion spray voltage, −4500 V; temperature, 550 °C. All samples were run in negative ion mode on a SCIEX QTRAP 5500 LC-MS/MS. The OTA standard solution was diluted with acetonitrile and the OTA concentration was monitored using MS. The mass spectrometer was optimized for maximum MRM signal intensity by injecting OTA standard samples directly into the mass spectrometer. In addition, enhanced product ion (EPI) experiments were performed in negative mode, with assistance from LightSight software. All EPI data were acquired under the following conditions: collision energy diffusion: −120 V; collision energy (CE), 29–45 V; scan range 80–700 *m*/*z*; scan rate: 10,000 Da/s; cycle time 50 msec. Data acquisition and processing were performed using AB Sciex Analyst software (version 1.5.1) and LightSight software (version 2.2.1).

### 4.10. Safety Evaluation of OTA Degradation Products

Human hepatoma HepG2 cells were cultured in Dulbecco’s modified eagle medium (DMEM) containing 10% fetal bovine serum at 37 °C in an incubator with 5% CO_2_ and 95% air. The OTA standard was dissolved in DMSO. HepG2 cells were treated with different concentrations of OTA, and the IC_50_ value was determined. The optimal concentration was selected for subsequent experiments, and the control group was exposed to DMSO only. The cells were treated with OTA, degradation products, and zymotic fluid to detect various cell parameters. Cell viability was assessed with the CCK-8 kit (Beyotime Biotechnology, Shanghai, China). The cell viability (*CV*) was calculated as follows:


$$CV=\frac{AS-AB}{AC-AB}\times 100\%$$

*AS* was the experimental group, *AC* was the control group, and *AB* was the blank group.

Cell morphology was observed under an inverted microscope. Reactive oxygen species (ROS) activity and mitochondrial membrane potential were assessed with the ROS Assay kit and the Mito-Tracker Red CMXRos kit (Beyotime Biotechnology, Shanghai, China).

### 4.11. Bio-Fermentation of A. niger to Degrade OTA in Wheat Bran

The wheat bran was crushed, sifted through a 30-mesh sieve, and mixed evenly. The wheat bran was collected. The crushed wheat bran was sampled, weighed, sub-packed in a 50 mL triangular conical flask, sealed with sealing film, and sterilized at 121 °C for 20 min. The sterilized wheat bran was transferred to the fermentation container, OTA was added to a concentration of 1 μg/g, and sterile water was added to the feed at a feed:water ratio of 1:2 (g:mL). Next, 15% FS-UV-21 spore suspension was added, stirred, and processed using constant temperature aerobic fermentation at 30 °C. The OTA content was determined after 5 d. The control group was 15% sterile water.

### 4.12. Statistical Analysis

GraphPad Prism 8.0 software (GraphPad Software Inc., La Jolla, CA, USA) was used for the statistical analysis of the data. Analysis of variance (ANOVA) and the Duncan multi range test were used to analyze the differences between the mean values. Values are shown as the mean ± SD of the three independent experiments. Significance was set at *p* < 0.01.

## Figures and Tables

**Table 1:** OTA degradation in wheat bran by *A. niger* FS-UV-21 fermentation (µg/mL ± SD).

Sample Names	OTA Content (μg/mL)		OTA Degradation Rate (%)
	Fermented 0 d	Fermented 5 d	
Control	1.142 ± 0.027	1.119 ± 0.049	2.01
FS-UV-21	1.112 ± 0.051	0.447 ± 0.064	59.84

## Data Availability

Not applicable.

## Supplementary Materials

The following supporting information can be downloaded at: https://www.mdpi.com/article/10.3390/toxins14050343/s1. Table S1. 18S rDNA sequencing results of FS-UV-21. The sequencing results were compared and analyzed on the NCBI website (https://blast.ncbi.nlm.nih.gov/Blast.cgi, accessed on 16 October 2020) to identify the species of the samples submitted for inspection. Generally, the species with a homology of more than 97% and the highest homology obtained by comparison with NCBI is judged as being the species of the submitted sample; Table S2: Information on OTA metabolic degradation products of OTA; Table S3: Gradient elution procedure; Figure S1: Phylogenetic tree of FS-UV-21.
