# Supplementary material for: Degradation of Ochratoxin A by a UV-Mutated Aspergillus niger Strain
Source: Toxins (Basel). 2022 May 16;14(5):343. doi: 10.3390/toxins14050343 (PMC9146908; doi:10.3390/toxins14050343)
Supplement: Supplementary file 1 [file toxins-14-00343-s001.zip › toxins-1702341-supplementary.pdf]

Degradation of Ochratoxin A by a UV-Mutated *Aspergillus niger* Strain

Supplementary Materials 4.7 Extraction and analysis of OTA degradation products

Sample preparation methods for metabolite analysis. The samples were divided into the experimental group and the control group. In the experimental group, *A. niger* FS-UV-21 was inoculated in PDB medium containing 1 μg/mL OTA, and the samples were cultured for 24, 36, and 48 h. *A. niger* FS-UV-21 was inoculated in PDB medium without OTA as the control group, and other experimental conditions were the same as the experimental group. After sampling, mycelium were collected and placed on ice, washed with 4ºC pre-cooled PBS buffer, and the buffer was removed through a vacuum filtration device. After repeating this 3 times, the mycelium was transferred to a clean 2 mL EP tube. These steps were all performed at 4ºC. The sample was then quenched in liquid nitrogen for 10 min, and 750 mL of isopropanol:acetonitrile:water (3:3:2) extract solution pre-cooled at −20ºC was added, and two stainless steel beads with a diameter of 2 mm and 3 mm were added at the same time. A crushing treatment was then carried out, with the crushing conditions being 2000 rpm for 30 s, for 8 cycles. Pre-cooled extract was added (750 mL), and the crushing process was repeated. The sample was centrifuged at 14000 rpm and 4ºC for 15 min, and the supernatant was extracted into a new 2 mL EP tube. The sample was evaporated to dryness with a vacuum freeze dryer, and 1 mL of chromatographic-grade acetonitrile solution was added for reconstitution, before waiting for detection. The sample was stored at −20°C before testing.

Table S1. 18S rDNA sequencing results of FS-UV-21. The sequencing results were compared and analyzed on the NCBI website (https://blast.ncbi.nlm.nih.gov/Blast.cgi) to identify the species of the samples submitted for inspection. Generally, species with a homology of more than 97% and with the highest homology obtained by the comparison on NCBI were judged as being the species of the submitted sample.

<table>
<tr><th>Detection method</th><th>Homology</th><th>Reference Species</th></tr>
<tr><td>CSQ</td><td>601/604 (99%)</td><td>Aspergillus niger</td></tr>
<tr><td colspan="3">GGCGGCCGGTTCCTCCGCCTTTTGATATGCTTAAGTTCAGCGGGTATCCCTACCT
GATCCGAGGTCAACCTGGAAAGAATGGTTGGAAAACGTCGGCAGGCGCCGGCC
AATCCTACAGAGCATGTGACAAAGCCCCATACGCTCGAGGATCGGACGCGGTG
CCGCCGCTGCCTTTCGGGCCCGTCCCCCCGGAGAGGGGGACGGCGACCCAACAC
ACAAGCCGGGCTTGAGGGCAGCAATGACGCTCGGACAGGCATGCCCCCCGGAA
TACCAGGGGGCGCAATGTGCGTTCAAAGACTCGATGATTCACTGAATTCTGCAA
TTCACATTAGTTATCGCATTTCGCTGCGTTCTTCATCGATGCCGGAACCAAGAGA
TCCATTGTTGAAAGTTTTAACTGATTGCATTCAATCAACTCAGACTGCACGCTTTC
AGACAGTGTTCGTGTTGGGGTCTCCGGCGGGCACGGGCCCGGGGGGCAGAGGC
GCCCCCCCGGCGGCCGACAAGCGGCGGGCCCGCCGAAGCAACAGGGTACAATA
GACACGGATGGGAGGTTGGGCCCAAAGGACCCGCACTCGGTAATGATCCTTCCG
CAGGTCCCCCTAACGGAAGGGCGGT</td></tr>
</table>

Table S2. Chemical information of metabolic degradation products of OTA.

**Compound**	**Q1(*m*/*z*)**	**Q3(*m*/*z*)**	**RT (min)**	**Peak area (%)**	**Biotransformation**	**Molecular formula**
P1	404.0	212.9	4.43	21.0	Demethylation + Oxidation	$C_{19}H_{18}ClNO_6$
P2	486.0	294.9	4.43	0.1	Di-Acetylation	$C_{22}H_{22}ClNO_8$
P3	416.0	224.9	4.46	0.1	Keto (Ox-2H)	$C_{19}H_{16}ClNO_7$

Table S3. Gradient elution procedure from LC-MS/MS analysis.

**Time**	Flow rate	Mobile phase（%）	
**（min）**	（mL/min）	A	B
0	0.35	95	5
1	0.35	95	5
6	0.35	5	95
8	0.35	5	95
8.10	0.35	95	5

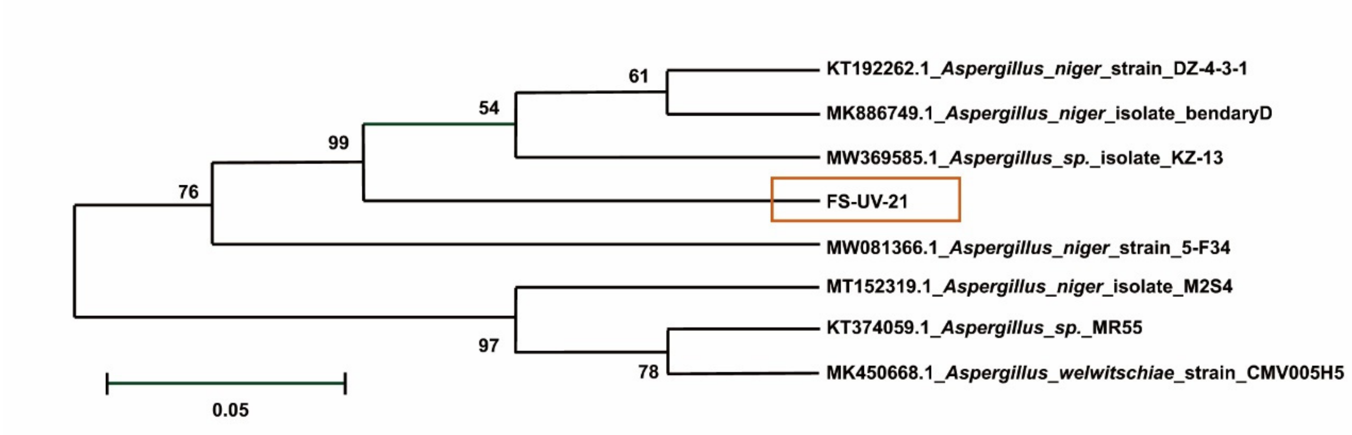

Figure S1. Phylogenetic tree of *A. niger* FS-UV-21 strain.
